# Effect of chondroitin sulfate modified polyethyleneimine on mediating oligodeoxynucleotide YW002 in the treatment of periodontitis†

**DOI:** 10.1039/d4ra00884g

**Published:** 2024-06-25

**Authors:** Xingyuan Qu, Qian Zhang, Chuang Zhang, Jichao Sun, Siyu Du, Chen Liang, Yabing Chen, Yi Zheng, Lei Wang

**Affiliations:** a Department of Periodontology, Hospital of Stomatology, Jilin University 1500 Tsinghua Road, Chaoyang District Changchun 130021 China zhengyi8304@jlu.edu.cn wang_lei99@jlu.edu.cn +86-0431-8879-6039 +86-139-4400-1891 +86-186-4498-6173; b Second Affiliated Hospital, Jinzhou Medical University 49 Shanghai Road, Guta District Jinzhou 121000 China; c Jilin Provincial Key Laboratory of Tooth Development and Bone Remodeling, Jilin University 763 Heguang Road, Chaoyang District Changchun 130021 China; d School of Pharmacy, Jilin Medical University 5 Jilin Street Jilin 132013 China; e Department of Chemistry, Northeast Normal University 5268 Renmin Street Changchun 130024 China

## Abstract

Purpose: In a previous study, we found that oligodeoxynucleotide (ODN) YW002 could induce the activity of alkaline phosphatase of early osteogenesis in human periodontal membrane stem cells, and downregulate the synthesis of nitric oxide in RAW 264.7 cells in the late inflammatory stage, laying the experimental foundation for the subsequent application of ODN YW002 in periodontitis. However, free ODN does not easily adhere to cells and is easily hydrolyzed by nuclease, so the immune effect of ODN is greatly reduced. Therefore, the nano-drug delivery system provides a method for efficient delivery and uptake of ODN. Methods: We synthesized a polyethyleneimine (PEI) modified chondroitin sulfate (CS) derivative (PEI-CS) *via* Michael addition to deliver ODN YW002. We aimed to evaluate whether PEI-CS could effectively deliver YW002 to RAW 264.7 cells and if it can regulate inflammation *in vitro*. PEI-CS/YW002 nanocomplexes were locally injected into a mouse periodontitis model, and the therapeutic effects were evaluated by microcomputed tomography (micro-CT) and hematoxylin–eosin (H&E) staining. Results: The results indicated that PEI-CS had good biocompatibility and could form a stable nanocomplex with YW002 at a mass ratio of 4 : 1. Moreover, PEI-CS could deliver YW002 into RAW 246.7 cells and markedly decreased the expression levels of interleukin (IL)-1β, IL-6 and tumor necrosis factor (TNF)-α. Histological evaluation and micro-CT scanning showed that PEI-CS/YW002 nanocomplexes effectively inhibited periodontitis and reduced alveolar bone resorption in mice. Conclusion: Our study has underscored the potential of PEI-CS/YW002 nanocomplexes as promising agents for the prevention and treatment of periodontitis due to their potent anti-inflammatory effects.

## Abstract 2

In a previous study, we found that oligodeoxynucleotide (ODN) YW002 could downregulate the synthesis of nitric oxide in RAW 264.7 cells, laying the experimental foundation for the subsequent application of ODN YW002 in periodontitis.

## Graphical abstract

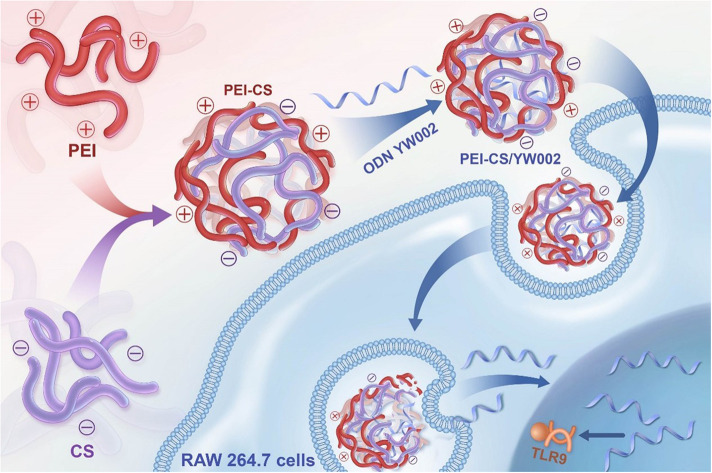

## 1. Introduction

Periodontitis refers to a prevalent oral disease that involves inflammation of the gingival tissue and loss of the alveolar bone.^1,2^ It is the primary cause of tooth mobility, displacement, or even loss. All these results significantly impact mastication, ingestion of food, and aesthetic aspects.^3–5^ The cause and development of periodontitis involve intricate factors. The Gram-negative anaerobic coccus, *Porphyromonas gingivalis* (*P. gingivalis*), is a key pathogenic bacterium implicated in the etiology and progression of periodontitis.^6^ Emerging evidence indicates that periodontitis is not exclusively characterized as a disease caused by pathogens; rather, it is increasingly recognized as a consequence of an imbalanced immune response, resulting in harm to the periodontal tissues.^7^ While periodontopathic bacteria serve as the initiators of the disease, it has been noted that the presence of inflammation significantly contributes to exacerbating tissue destruction. Therefore, considering the evolving understanding of periodontitis, our treatment approach should focus on exploring strategies to effectively inhibit inflammatory processes.

Nowadays, there has been significant interest in the control of immune response to decrease inflammation, regulate the environment that leads to bone loss, and promote natural bone formation.^8^ Unmethylated nucleotide core motif sequences, known as oligodeoxynucleotides (ODN), have a crucial impact on modulating the immune response. In comparison to conventional small molecule drugs, ODN exhibits distinctive advantages and holds promising prospects for applications. Numerous studies have demonstrated the extensive applicability of ODN YW002 in inhibiting inflammation and modulating immune responses. YW002 is a type C ODN with both type A and B effects, leading to the activation of plasmacytoid dendritic cells for robust production of type I interferon and stimulation of B cells to generate antibodies. This multifaceted mechanism plays a pivotal role in suppressing inflammation.^9^ In our previous research, we successfully demonstrated the significant inhibition of inflammatory factor gene expression and the effective suppression of osteoclast formation and development by YW002.^10^

The most significance in the clinical application of ODN lies in safeguarding it against DNase degradation and facilitating its delivery to the toll-like receptor (TLR)^9^ on target cells. However, the stability of ODN in terms of nuclease mediated degradation and its delivery efficiency to target organs are insufficient, thereby significantly restricting its extensive clinical utilization. The chemical modification of ODN represents an efficacious technique for safeguarding against degradation by DNase. However, there have been a number of reports of serious side effects which are due to the modification of the DNA backbone. The recent years have witnessed the conduction of extensive research on nanoparticle-based delivery systems for ODNs. It is imperative to develop a safe delivery system for ODN in nanoparticles. Polyethyleneimine (PEI) is the most commonly used gene vector at present, but it has biological toxicity. To address this limitation, extensive research has been conducted on the chemical modification of PEI to enhance its transfection efficiency and/or mitigate its cytotoxicity, with particular emphasis on grafting hydrophobic moieties. The present study demonstrates a successful construction of chondroitin sulfate (CS)-modified polyethyleneimine (PEI). This novel construct demonstrates reduced cytotoxicity, improved capacity for targeting tumors, and comparable efficiency in transfection when utilizing pEGFP-C1 as a model, similar to Lipofectamine 2000.^11^ Yw002-s is a thiolated variant of YW002. Thiolation enhances the stability and immunostimulatory activity of ODN, although it may increase the risk of adverse reactions, leading to potential toxicity and higher synthesis costs.

**Fig. 1:**
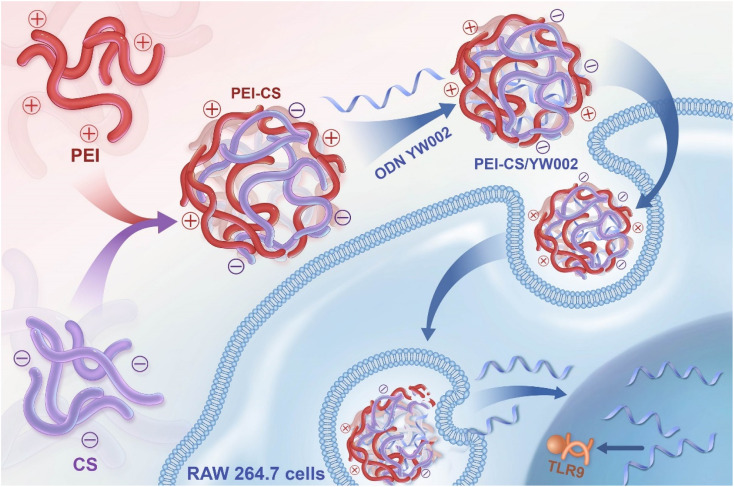
Synthesis of PEI-CS and delivery of ODN YW002 by PEI-CS.

In this study, a carrier PEI-CS was synthesized for the delivery of YW002 by chemically conjugating CS to PEI *via* Michael addition. We aimed to evaluate their therapeutic effects on *P. gingivalis*-derived lipopolysaccharide (*P.g.*-LPS)-induced inflammation and periodontitis in mice (Fig. 1).

## 2. Results

### 2.1. Synthesis and characterization of PEI-CS

**Fig. 2:**
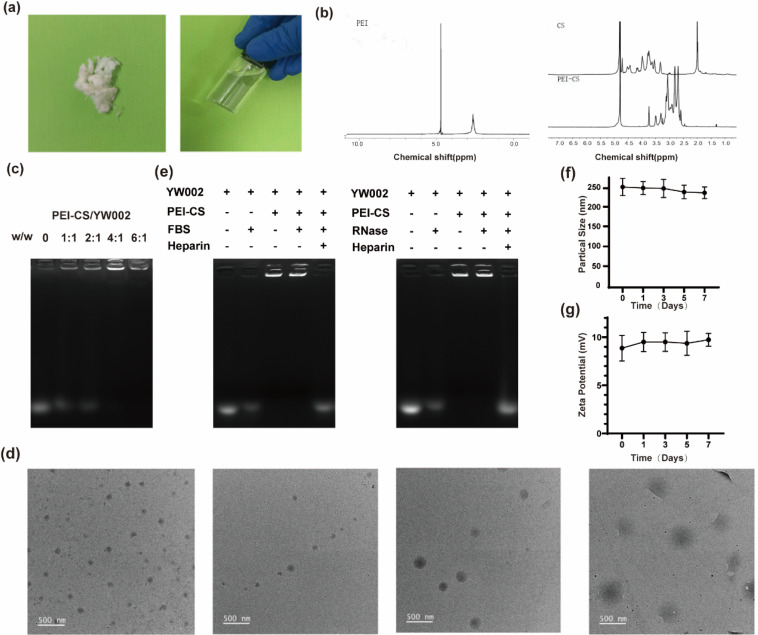
Characterizations and stability tests of PEI-CS/YW002 nanocomplexes. (a) The synthesis of PEI-CS. (b) Structure of PEI, CS and PEI-CS characterised by ^1^H-NMR. (c) Agarose gel retardation assays of PEI-CS/YW002 nanocomplexes at different w/w ratios of 0, 1 : 1, 2 : 1, 4 : 1, 6 : 1. (d) The TEM images of PEI-CS/YW002 nanocomplexes at various w/w ratios of 0, 2 : 1, 4 : 1, 6 : 1. (e) Detection of protective ability of PEI-CS carrier for YW002. (f) Size of PEI-CS/YW002 at 4 °C over a period of 7 days. (g) Zeta potential of PEI-CS/YW002 at 4 °C over a period of 7 days.

The synthesis of PEI-CS was achieved *via* the Michael addition method.^12^ The structure of CS, PEI, and PEI-CS were characterized by ^1^H nuclear magnetic resonance (^1^H-NMR) (Fig. 2a). The results suggested that the CS, PEI and PEI-CS samples all had a methyl peak at 4.75 ppm, that was, the acetylamino group of CS, while the PEI-CS spectrum showed a hydrogen proton peak in the polyethyleneimine-CHCH_2_NH- structure at the chemical shift *δ* 2.5–3.0 ppm, which was the result of the shift of the PEI peak from the 2.5 ppm during the reaction. It was further proved that CS reacted with PEI and PEI-CS complex was successfully constructed. The agarose gel retardation assay was employed to examine the binding capacity of PEI-CS and YW002. The retardation of YW002 was observed to be fully achieved when using PEI-CS at a mass ratio exceeding 4 : 1, suggesting that stable nanocomplexes of YW002 could be formed through electrostatic interaction with PEI-CS (Fig. 2b). The optimal particle size and positively charged profile of PEI-CS/YW002 nanocomplexes facilitated their efficient endocytosis and transfection. As the polymer/YW002 ratio increased, nanocomplexes ranged from 139.73 ± 0.73 to 346.23 ± 0.73 nm in size, and zeta potential increased from −17.40 ± 3.33 mV at a ratio of 2 : 1 up to +21.5 ± 5.82 mV at 8 : 1 (Table 1). To further characterize PEI-CS/YW002 nanocomplexes, we applied TEM. According to the results (Fig. 2d), we could find that PEI-CS/YW002 nanocomplexes with different mass ratios had uniform size of about 200 nm and single particle size distribution.

**Table 1:** Characterization of particle size and zeta potential of PEI-CS/YW002 nanocomplexes at various ratios

Sample	Particle size (nm)	Zeta potential (mV)
2 : 1	346.23 ± 0.73	−17.40 ± 3.33
4 : 1	240.03 ± 0.38	+9.10 ± 1.78
6 : 1	178.67 ± 0.81	+14.03 ± 1.03
8 : 1	139.73 ± 0.73	+21.50 ± 5.82

In order to investigate the stability of PEI-CS/YW002 nanocomplexes, the samples were placed at 4 °C for 7 days and sampled at 1, 3, 5 and 7 days respectively, the results showed that there was no major change in the particle size of the samples in each group, indicating that the resulting PEI was stable and could be stored for a long period of time (Fig. 2f). The zeta potential of each group of samples was examined, and the results showed that there was no significant change in the potential of each group, and the potential results also showed that the PEI-CS/YW002 had high stability (Fig. 2g).

### 2.2. Evaluation of the protective effect of PEI-CS on YW002

Since YW002 is easily degraded *in vivo*, the vector is crucial for the transfection efficiency of YW002, especially under *in vivo* conditions, so we evaluated the protective effect of PEI-CS on YW002 using gel blocking assay. The PEI-CS/YW002 nanocomplexes were incubated with 0.05 mg mL^−1^ RNase A and 50% FBS, respectively, and then the nanocomplexes were treated with heparin at a concentration of 10 mg mL^−1^, and then detected by 1% agarose gel electrophoresis, and the results are shown in Fig. 2e. The treatment of FBS or RNase A could easily degrade the free YW002, while the PEI-CS/YW002 nanocomplex could still observe clear bands after FBS or RNase A treatment, indicating that PEI-CS could protect YW002 from degradation by RNase A and FBS. Therefore, the vector PEI-CS can effectively stabilize and protect YW002 from the degradation effects of nuclease and serum, which is expected to enhance the half-life of YW002 during transfection and *in vivo* application, and thus obtain good therapeutic effects.

### 2.3. The biocompatibility of PEI-CS/YW002 nanocomplexes

**Fig. 3:**
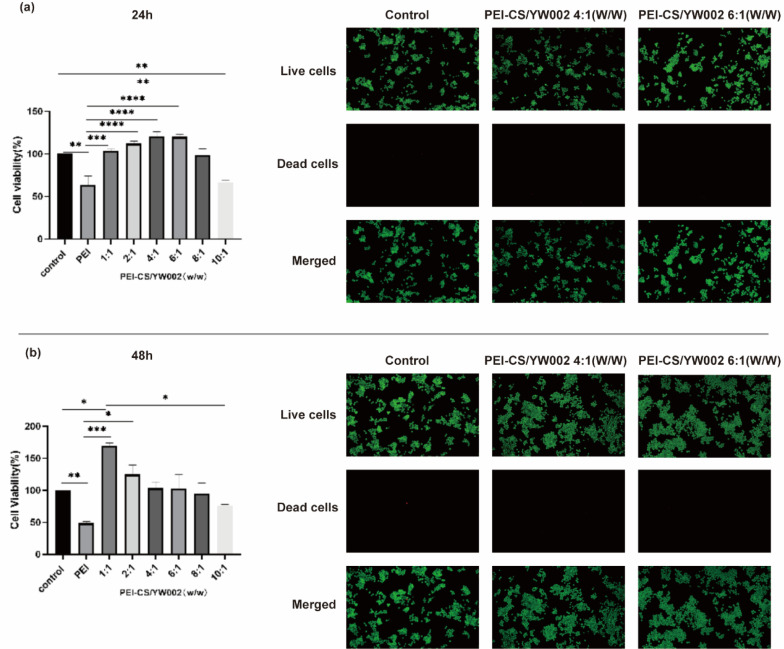
The biocompatibility of PEI-CS/YW002 nanocomplexes (a) the viability of cells cultured with PEI-CS/YW002 nanocomplexes after 24 hours. (b) The viability of cells cultured with PEI-CS/YW002 nanocomplexes after 48 hours. Green indicates the presence of viable cells, while red represents non-viable cells. The data were presented as the mean value ± SD of three experiments. *: *p* < 0.05, **: *p* < 0.01, ***: *p* < 0.001, ****: *p* < 0.0001.

To assess the potential cytotoxic effects of PEI-CS/YW002 nanocomplexes, RAW 264.7 cells were exposed to varying w/w ratios of complexed PEI-CS/YW002 and a control group treated with PEI alone (Fig. 3). We could see the cell viability of PEI-CS/YW002 nanocomplexes was superior to PEI, while that of PEI was less than 50%. When the mass ratio was below 8 : 1, no significant toxicity to cells was observed (*p* > 0.05). However, at a ratio of 10 : 1, there was a notable increase in cytotoxicity (*p* < 0.01). The complex exhibited a significant increase in the value of RAW 264.7 cells when the mass ratio was 4 : 1 and 6 : 1, compared to other groups (*p* < 0.05). However, we found that when mass ratio was 10 : 1, PEI-CS/YW002 nanocomplexes still did not show toxicity when cultured for 24 hours. The reason we considered was that the time was short and the toxicity had not yet been shown. The cell distribution was uniform under inverted fluorescence microscopy, and there was a progressive rise in the count of viable cells as the incubation period extended. The percentage of deceased cells was less than 1% at both the 24 hour and 48 hour time points, as observed using a low magnification (×10) field of view. Obviously, PEI-CS/YW002 nanocomplexes based on the coacervation of electrostatic interactions showed good biocompatibility, which provides a basis for their subsequent use.

### 2.4. The ability of cellular uptake of PEI-CS/YW002 nanocomplexes

**Fig. 4:**
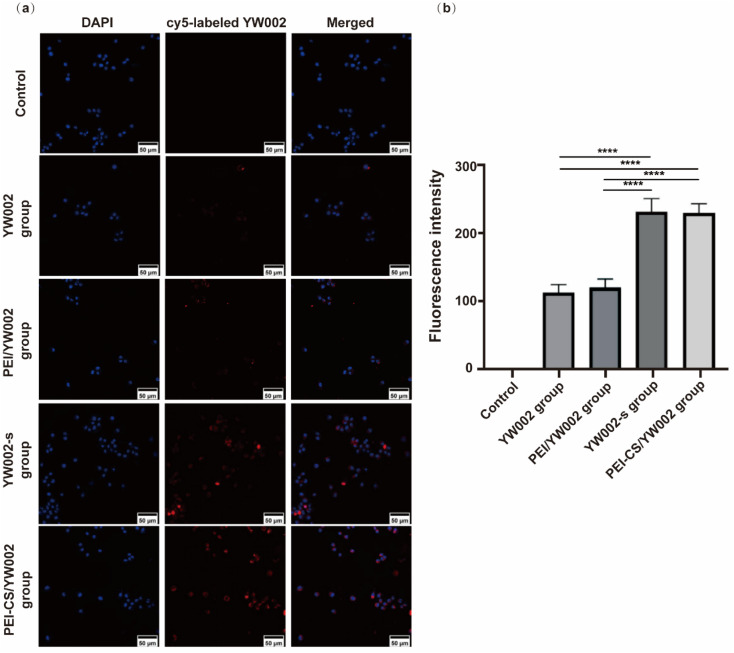
The effects of endocytosis and uptake of PEI-CS/YW002 nanocomplexes. (a) The CLSM images of RAW 264.7 cells exposed to PEI-CS/cy5-labeled YW002 nanocomplexes at a mass ratio of 4 : 1 for 4 h. Blue: nuclei (4,6-diamidino-2-phenylindole solution (DAPI)); red: cy5-labeled YW002/YW002-s. The scale is 50 μm. (b) Semi-quantitative detection of CLSM fluorescence intensity. ****: *p* < 0.0001.

To monitor the internalization of YW002 into RAW 264.7 cells mediated by PEI-CS, YW002 and YW002-s were labeled with Cy5 fluorescent dye for visualization using Confocal laser scanning microscope (CLSM) (Fig. 4). After a 4 hour incubation period, a significantly stronger red fluorescence was observed in the perinuclear regions of the cytoplasm of RAW264.7 cells transfected with YW002 through PEI-CS compared to the control, free YW002 and PEI/YW002 group indicating that PEI-CS achieved higher efficiency in facilitating YW002 transfection. In addition, we could observe that the fluorescence intensity of YW002-s group was similar to that of PEI-CS/YW002 group (*p* > 0.05).

### 2.5. The effect of inflammation markers expression of PEI-CS/YW002 nanocomplexes

**Fig. 5:**
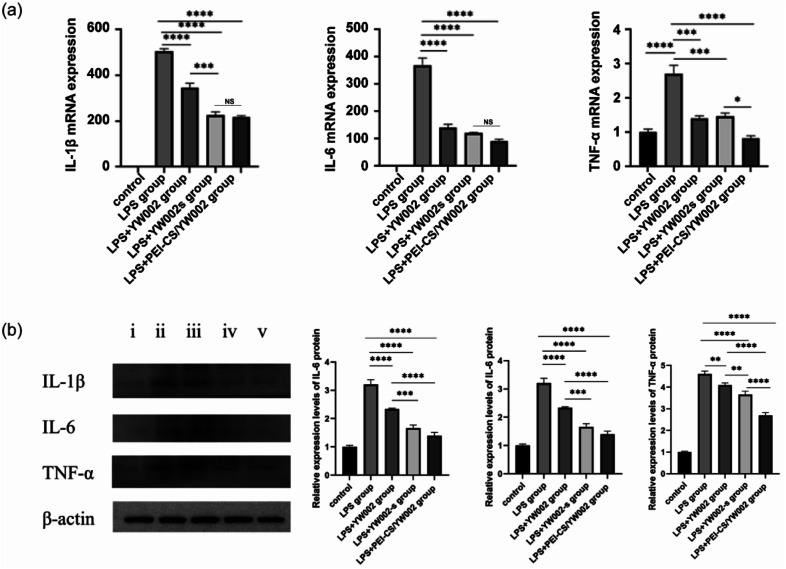
Inflammation factor characteristics of PEI-CS/YW002 nanocomplexes. (a) IL-1β, IL-6 and TNF-α mRNA expression. (b) IL-1β, IL-6 and TNF-α protein expression. (i) Control group, (ii) LPS group, (iii) LPS + YW002 group, (iv) LPS + YW002-s group, (v) LPS + PEI-CS/YW002 group. *: *p* < 0.05, **: *p* < 0.01, ***: *p* < 0.001, ****: *p* < 0.0001, NS: no significance.

The expression of the gene level was assessed using the real-time (RT)-PCR, with β-actin as a reference housekeeping gene. To further investigate the anti-inflammatory effects of YW002 on RAW 264.7 cells, we quantified the mRNA and protein expressions of inflammation-related genes using RT-PCR and western blot analysis. RT-PCR results (Fig. 5a) showed that the expression of these inflammatory cytokines decreased significantly after PEI-CS/YW002 and YW002-s treatment (*p* < 0.05). The expression of inflammatory cytokines following treatment with YW002 alone did not exhibit a statistically significant difference compared to the periodontitis group (*p* > 0.05). The results of our western blot (Fig. 5b) and RT-PCR (Fig. 5a) experiments showed a similar trend in the expression of inflammatory cytokines. In summary, the utilization of PEI-CS/YW002 nanocomplexes demonstrates a significant reduction in the expression of inflammatory factors, thereby attenuating the progression of inflammation. This novel approach holds promise for future therapeutic interventions targeting periodontitis.

### 2.6. CT evaluation of the effect of PEI-CS/YW002 nanocomplexes on mice alveolar bone

**Fig. 6:**
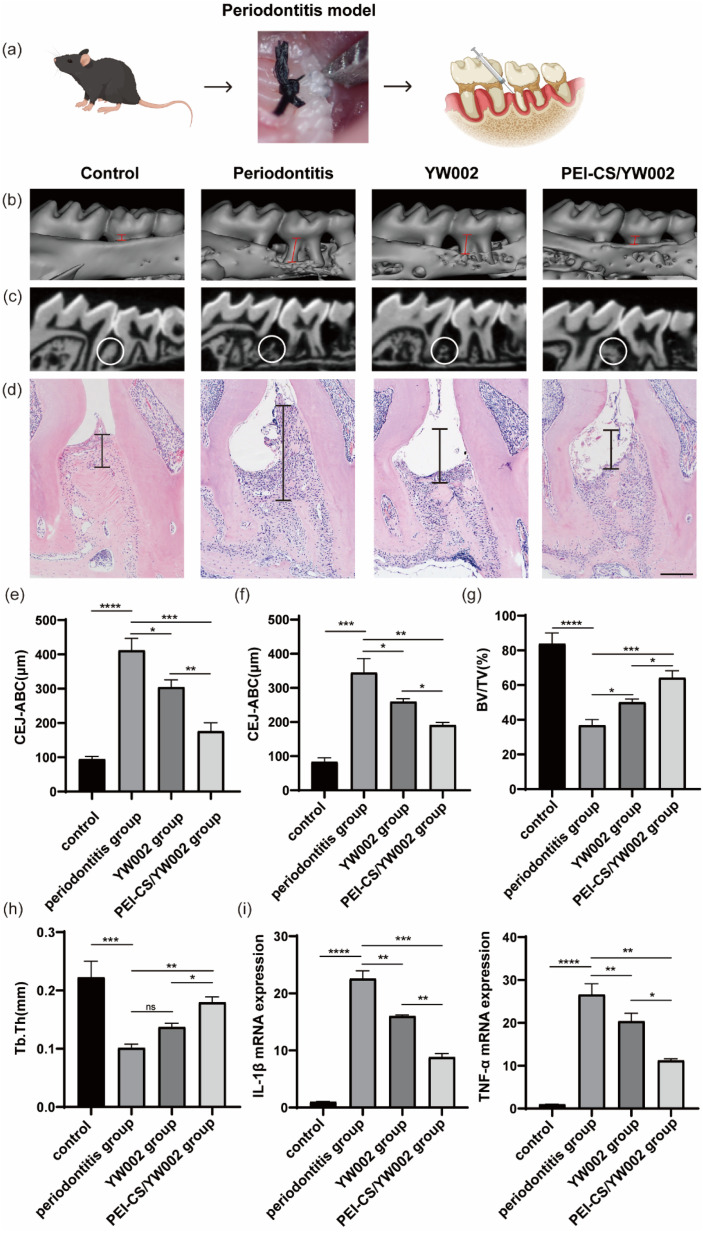
*In vivo* therapeutic and anti-inflammatory effects of PEI-CS/YW002 nanocomplexes. (a) Schematic diagram of modeling periodontitis mice and drug administration. (b) 3D reconstructions of maxillae of control, periodontitis group, YW002 group, PEI-CS/YW002 group (*n* = 3 per group) were generated by micro-CT. The vertical line extends from the CEJ to the ABC. (c) Representative sagittal micro-CT slices. The circled region represents the subsequent analysis area for evaluating alveolar bone parameters. (d) The histological sections of the periodontium from each group were visualized using H&E staining. A vertical line was drawn from the CEJ to the ABC, and the distance between CEJ and ABC was measured in each microscope field of view. Scale bar = 100 μm. (e) Statistical analysis of CEJ-ABC distance by micro-CT in each group (*n* = 3 per group). (f) Statistical analysis of CEJ-ABC distance by H&E staining in each group (*n* = 3 per group). (g) Quantitative analysis of BV/TV determined by micro-CT images. (h) Quantitative analysis of Tb.Th determined by micro-CT images. (i) IL-1β and TNF-α mRNA expression*: *p* < 0.05, **: *p* < 0.01, ***: *p* < 0.001, ****: *p* < 0.0001.

Given that periodontitis is characterized by alveolar bone resorption, our study aimed to assess the effects of PEI-CS/YW002 nanocomplexes on the process of alveolar bone remodeling. To ensure consistency of measurement criteria, we adjusted the viewing angle of all micro-CT images so that all tooth cusps were in the same plane and the occlusal plane was not visible from the buccal side. The micro-CT scans (Fig. 6b) showed that the periodontitis group showed significantly lower levels of alveolar bone, with a recessed alveolar bone between the first and second molars, compared to the control group. The proximal buccal and distal buccal roots of the second molar were revealed, and the alveolar bone level was elevated in the YW002 group, but much lower than that in the PEI-CS/TW002 group. The extent of alveolar bone loss can be determined by measuring the vertical space between the cementoenamel junction (CEJ) and the tip of the alveolar bone (ABC). ImageJ measured significant differences in CEJ-ABC values between the four groups (Fig. 6e and f). It was found that the CEJ-ABC distance contracted by 26.00% (*P* < 0.05) and 55.26% (*P* < 0.001) in the YW002 group and the PEI-CS/YW002 group, respectively, compared with the periodontitis group in 3D images, and by 24.8% in the YW002 group and the PEI-CS/YW002 group, respectively, compared with the periodontitis group in the HE sections (*P* < 0.05) and 44.55% (*P* < 0.01), indicating that PEI-CS/YW002 significantly promoted alveolar bone regeneration. Further examination of the region of interest located between the first and second molars revealed a significant increase in BV/TV by 13.23% (*p* < 0.05) and 27.39% in the YW002 and PEI-CS/YW002 groups, respectively, when compared to the periodontitis group (Fig. 6g) (*p* < 0.05). Additionally, there was an observed elevation of Tb.Th by 0.078 mm in the PEI-CS/YW002 group compared to the periodontitis group (Fig. 6h) (*p* < 0.01). These results suggest that PEI-CS/YW002 nanocomplexes infusion can alleviate experimental periodontitis and reduce alveolar bone loss in mice.

### 2.7. Anti-inflammatory properties of PEI-CS/YW002 nanocomplexes *in vivo*

The sustained inflammatory response is an abnormal repair mechanism for periodontitis-induced injury. Therefore, we further confirmed the role of PEI-CS/YW002 nanocomplexes in attenuating periodontitis in mice. HE staining results (Fig. 6d) showed that the collagen fibers in the periodontal supporting tissues of the healthy mice were neatly aligned, with smooth alveolar bone surfaces, and gingival epithelium in the form of long, slender strips with intact structures. On the contrary, the normal epithelial structure of the periodontium was disrupted in the periodontitis group, leading to an extension of combined epithelium towards the root surface and formation of a deep periodontal pocket. The collagen fibers present in the epithelium of the gingival sulcus and connective tissue beneath it exhibited signs of edema and degeneration, resulting in a disorganized tissue structure with significant infiltration of inflammatory cells. Inflammatory cell infiltration was reduced after treatment with PEI-CS/YW002 nanocomplexes, and the height of alveolar bone was increased. The gene expression of IL-1β and TNF-α in periodontal tissues of mice with periodontitis was significantly upregulated compared to normal mice, while the gene expression of IL-1β and TNF-α decreased in the YW002 group compared to the periodontitis group, with an even more significant decrease observed in the PEI-CS/YW002 group (Fig. 6i).

### 2.8. The biocompatibility of PEI-CS/YW002 nanocomplexes *in vivo*

**Fig. 7:**
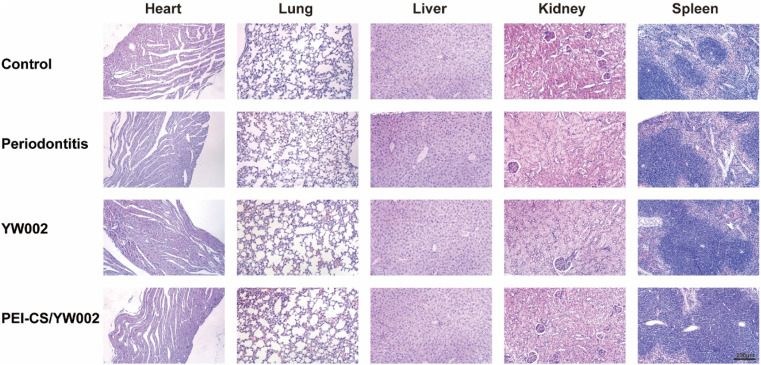
Biocompatibility of PEI-CS/YW002 nanocomplexes *in vivo*.

The oral condition of the mice was examined before each injection of the drug, and except for the periodontal tissues, the mucous membranes of the palate, buccal, and pharyngeal regions of the mice showed no abnormalities. To demonstrate the *in vivo* biocompatibility of PEI-CS/YW002 nanocomplexes, we conducted HE staining analysis on the hearts, lungs, livers, kidneys, and spleens of four distinct groups of mice. The results indicated no notable distinctions among these groups (Fig. 7).

## 3. Discussion

The chronic inflammation of periodontal tissue, known as periodontitis, can result in progressive degradation of the periodontal ligament and alveolar bone, loss of periodontal attachment, tooth mobility and displacement, and ultimately tooth loss. Oral health education and guidance, gingival clean treatment, scaling and root planing (SRP) is the current clinical for periodontitis common and effective treatment, but the treatment by tooth anatomical conditions, such as root bifurcation lesions, periodontal pocket depth, usually cannot completely eliminate plaque microbial.^13^ Moreover, the strength of host immune response is the key factor for the outcome of periodontitis.^14^

At present, it has been recognized that periodontitis is a multi-factor disease, and dental plaque is the initial factor causing periodontitis. However, the host's immune response can also damage local periodontal tissue. Therefore, drug therapy is often used to reduce periodontal pathogens in dental plaque and prevent the immune response when microorganisms invade or spread.^15^ In recent years, based on the efficacy and mechanism of ODN, many scholars have used ODN and ODN nanoparticles in the treatment of periodontitis, and made some progress.

ODN is a TLR agonist and an immunoprotective agent against bacterial, viral and protozoan infections. In our previous study, we found that the selected ODN YW002 could significantly inhibit the gene expression levels of inflammatory factors at different stages and significantly reduce the inflammatory response to the host.^10^ ZHAO^16^ found that moderate application of ODN YW002 helped to suppress myocarditis infection induced by coxsackievirus B3. However, YW002 is a negatively charged molecule with poor stability *in vitro*, and cannot spread freely on the cell membrane like lipophilic small molecule drugs, and its therapeutic target is in the cell, so its precise delivery to the target cell is the main challenge limiting the therapeutic development of ODN YW002. Whereas, the negative charge on ODN surface affects the modification effect, and chemical modification has toxic side effects.^17^ Therefore, polymer gene carriers, such as PEI, are used as preferred nanoscale drug carriers due to their excellent efficiency of phagocytosis, passive tumor targeting, high encapsulation rate, and wide therapeutic drug delivery capabilities.^18^ Although PEI is a gold standard for nonviral gene delivery vectors, its positively-charged surface characteristics results in its inherent cytotoxicity.^19,20^ This limits its development as an efficient gene delivery carrier. CS is an important component of extracellular matrix (ECM) in animals and humans. CS has important biological characteristics and plays a key role in all aspects of tissue and cell physiology. In recent years, it has been found that CS can promote cartilage regeneration, anti-inflammation,^21^ regulate blood lipids^22^ and anticoagulant activity.^23^

In this study, we used Michael addition method to graft CS onto PEI to prepare PEI-CS nanocomplex in order to reduce the cytotoxicity of PEI itself and effectively deliver ODN YW002. In order to determine whether the synthesis of the sample was successful, we performed a ^1^H-NMR test on the PEI-CS sample, and the map results showed that it was displayed at *δ* = 2.5–3.0 ppm The chemical shift of PEI methylene proton characteristics showed the characteristic hydrogen signal of CS at *δ* = 4.5–5.0 ppm. It can be seen that CS and PEI are successfully crosslinked to form PEI-CS nanomaterials (Fig. 2a and b). Many studies had shown that PEI-CS realizes the direct and efficient targeted delivery of drugs to the acting site, which provided a method for efficient delivery and uptake of ODN. The efficient gene transfection was achieved in Lo's previous study through the utilization of PEI-CS, which facilitated CD44-mediated endocytosis and enhanced the cellular uptake of CP/pDNA polyplexes in addition to clathrin-mediated endocytosis.^24^ Moreover, PEI-CS was found to significantly enhance the green fluorescence in PC-3 cells, suggesting its superior ability to facilitate miR-34a transfection.^25^ It can be seen that PEI-CS is a potential good gene delivery vector. Moreover, one study found that the gene vector for CS-modified PEI was successfully constructed using pEGFP-C1 plasmid as a model. PEI-CS preparation reduced cytotoxicity and enhanced tumor targeting, and the transfection efficiency was comparable to compared with the available Lipofectamine 2000® transfection reagent.^11^ We also found that PEI-CS complex could not only reduce the toxicity of PEI, but also enhance the delivery efficiency of PEI, and had the biological characteristics of CS at the same time. Through the characterization and detection of the complex, it was proved that its successful synthesis, but also provided a theoretical basis for follow-up experiments.

In Table 1 and Fig. 2d, when the PEI-CS/YW002 nanocomplexes were electrostatically bound and had a mass ratio of 4 : 1, the potential was normalized, that was, +9.10 ± 1.78 mV, and its particle size was 240.03 ± 0.38 nm, which indicated PEI-CS/YW002 nanocomplexes had good particle size and zeta potential. As we all know, the positive potential helps nanocomplexes to cross the cell membrane and enter the cell, and the appropriate particle size can promote the uptake of nanocomplexes by cells. In addition, in the experiment to detect the loading capacity of PEI-CS to YW002 (Fig. 2c), we found that PEI-CS was not associated with YW002 when the mass ratio is more than or equal to 4 : 1, PEI-CS could effectively block YW002 to the well load. The results of cell viability experiments (Fig. 3) showed that PEI-CS/YW002 nanocomplexes had good biocompatibility, and PEI-CS was a suitable gene carrier compared to PEI. The aforementioned experimental outcomes may offer valuable insights for optimizing the utilization of drug nanocarriers to achieve maximum effectiveness. Consequently, a mass ratio of 4 : 1 was ultimately chosen for subsequent investigations.

The cellular uptake of PEI-CS/YW002 nanocomplexes was studied by CLSM, which is a method to determine whether the nanocomplexes are taken up by cells. The position of the nucleus with DAPI staining could further determine the position of the cells. YW002 and YW002-s labeled with Cy-5 could observe cell uptake of YW002 (Fig. 4a). Compared with the blank control group and the free YW002 group, the red fluorescence of the cells of the PEI-CS/YW002 group was enhanced and mainly in the perinuclear region of the cells, indicating that PEI-CS could achieve higher YW002 transfection efficiency. Studies had shown that physicochemical properties such as particle size, surface potential, solubility, and surface function affect the uptake and removal of nanocomplexes by cells.^26^ Combining particle size and potential data, we further demonstrated the importance of adjustable particle size and surface potential for effective targeted delivery. In addition, the results of Fig. 4 showed that the fluorescence intensity of the YW002-s group is similar to that of the PEI-CS/YW002 group, which proved that the nanocomplexes delivery system could achieve the same effect as chemical modification as an effective protection against ODN technology degraded by nucleases.

The Gram-negative oral anaerobe *P. gingivalis*, implicated in the pathogenesis of periodontitis, contributes to the induction of elevated levels of inflammatory cytokines, including interleukin (IL)-1β, IL-6, and tumor necrosis factor (TNF)-α.^6^ These inflammatory cytokines are important for host resistance to bacterial invasion and are one of the steps in inflammatory regulation. In general, it is postulated that the pro-inflammatory cytokines IL-1β, IL-6, and TNF-α exert a pivotal role in orchestrating osteoclast activation leading to subsequent bone resorption.^27^ These inflammatory cytokines have the potential to enhance osteoclast differentiation, thereby facilitating alveolar bone resorption and destruction. In the present study, elevated levels of IL-1β, IL-6, and TNF-α were observed in the periodontitis group, indicating an inflammatory response mediated by inflammatory cells during disease progression (Fig. 5). Consistent findings from various studies have consistently demonstrated heightened proinflammatory cytokine levels within inflamed periodontal tissues.^28,29^ On the contrary, the administration of YW002-s and PEI-CS/YW002 effectively suppressed inflammatory responses, leading to a reduction in bone destruction in experimentally induced periodontitis. After co-culture with RAW 264.7 cells with only free YW002, IL-1β, IL-6 and TNF-α were still elevated. The difference in the LPS stimulation group was not obvious, indicating that free YW002 was insufficient to achieve effective transmission and failed to achieve effective anti-periodontitis effect. Instead, we observed the levels of IL-1β, IL-6, and TNF-α in YW002-s group and PEI-CS/YW002 group were lower than that in LPS-stimulated group, suggesting that both could inhibit the inflammatory response and achieve anti-inflammatory effects, which was also consistent with the previous results of our group.^10^ In the mouse periodontitis model, the application of PEI-CS/YW002 group could significantly improve the alveolar bone height of the mice, and the BV/TV and Tb.Th values were significantly increased (Fig. 6). Therefore, PEI-CS is a highly efficient carrier that can improve the transfection efficiency of YW002, and the effect is obvious when the mass ratio of PEI-CS/YW002 is 4 : 1. PEI-CS/YW002 nanocomplexes could be of great significance for inhibiting periodontal inflammation and have a wide range of application prospects.

## 4. Conclusion

In summary, the PEI-CS carrier was successfully synthesized through Michael addition, enabling efficient delivery and endocytosis of YW002 transfection. In addition, PEI-CS mediated ODN YW002 showed good biocompatibility, low toxicity and good anti-inflammatory effect may *in vitro* experiments when the mass ratio of PEI-CS/YW002 is 4 : 1. In conclusion, the utilization of PEI-CS as a nanocarrier ligand demonstrates promising potential for efficient YW002 delivery, thereby offering a viable therapeutic approach for addressing periodontitis in future treatment strategies.

## Materials and methods

Detailed methods are provided in the ESI† of this paper.

## Ethical statement

This study and included experimental procedures were approved by the institutional animal care and use committee of College of Basic Medical Sciences, Jilin University, China (Ethical approval number: SYXK2023-0010). All animal housing and experiments were conducted in strict accordance with the institutional guidelines for care and use of laboratory animals.

## Data availability

The data presented in this study are available on request from the corresponding authors.

## Author contributions

Xingyuan Qu: project administration, writing—original draft. Qian Zhang: conceptualization, funding acquisition. Chuan Zhang: methodology, formal analysis. Jichao Sun: visualization. Siyu Du: visualization, formal analysis. Chen Liang: visualization, data curation. Yabing Chen: formal analysis. Yi Zheng: writing—review and editing, resources, supervision, validation. Lei Wang: resources, supervision, validation. All authors have read and agreed to the published version of the manuscript.

## Conflicts of interest

The authors declare no conflict of interest. The sponsoring institute had no role in the design, execution, interpretation, or writing of the study.

## Supplementary Material

RA-014-D4RA00884G-s001

RA-014-D4RA00884G-s002

RA-014-D4RA00884G-s003
