# Supplementary material for: Effect of chondroitin sulfate modified polyethyleneimine on mediating oligodeoxynucleotide YW002 in the treatment of periodontitis
Source: RSC Adv. 2024 Jun 25;14(28):20328–38. doi: 10.1039/d4ra00884g (PMC11197841; doi:10.1039/d4ra00884g)
Supplement: RA-014-D4RA00884G-s001 [file RA-014-D4RA00884G-s001.pdf]

Electronic Supplementary Material (ESI) for RSC Advances.
This journal is © The Royal Society of Chemistry 2024

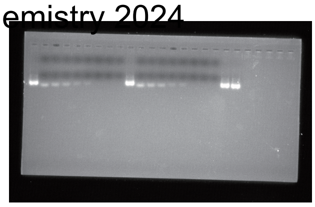

Fig 2c Agarosegel retardation assays of PEI-CS YW002 nanocomplexes at different ww ratios

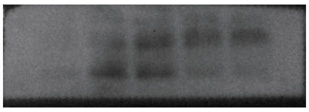

Fig 4b IL-1β

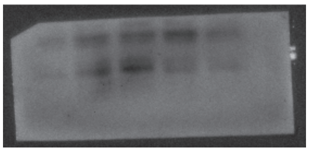

Fig 4b TNF-α

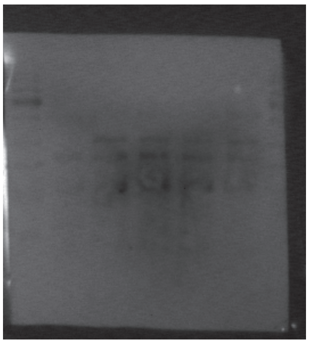

Fig 4b IL-6

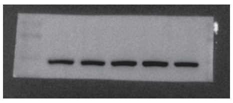

Fig 4b β-actin

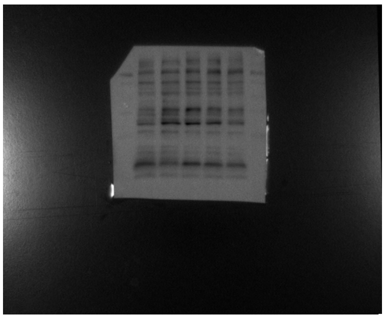

IL-1β 2

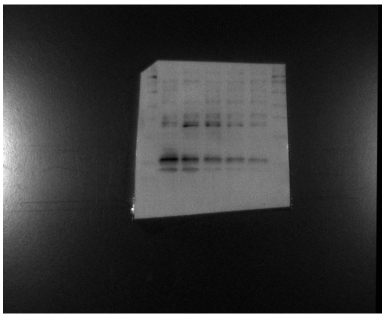

TNF-α 2

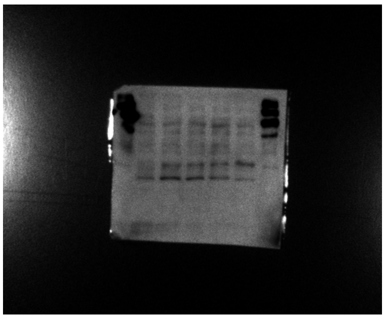

IL-6 2

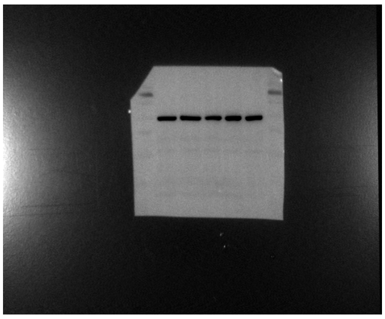

IL-1β 2 β-actin

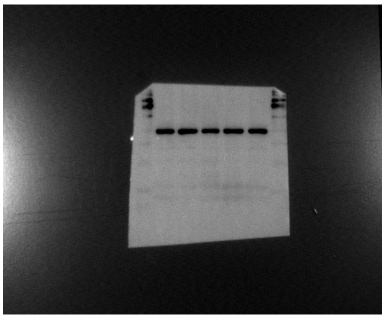

TNF-α 2 β-actin

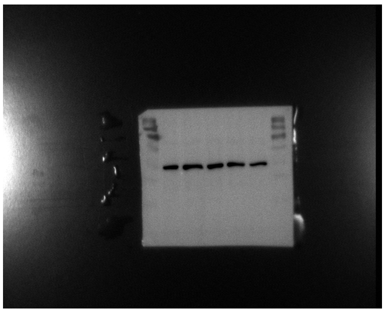

IL-6 2 β-actin

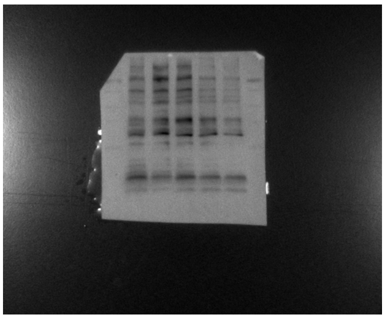

IL-1β 3

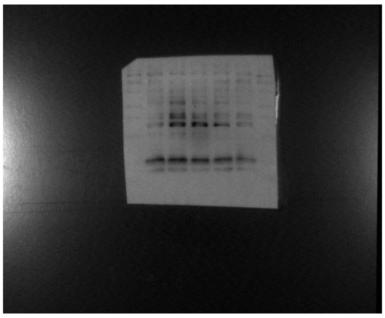

TNF-α 3

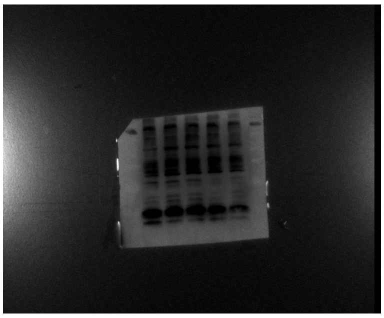

IL-6 3

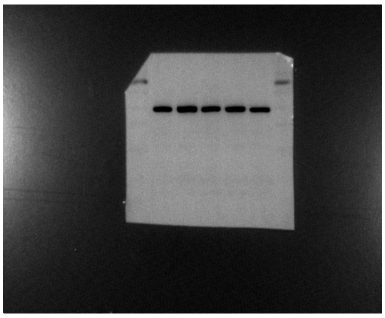

IL-1β 3 β-actin

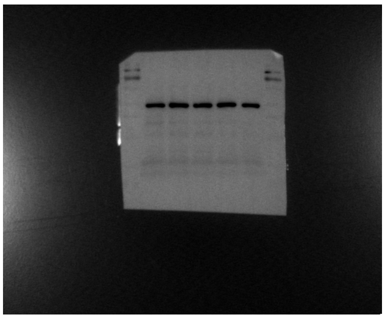

TNF-α 3 β-actin

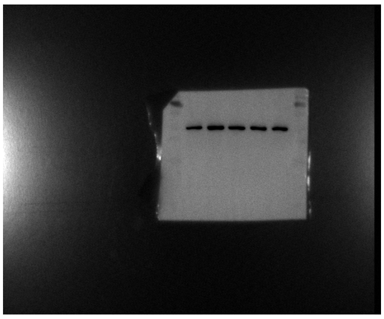

IL-6 3 β-actin
