# Supplementary figures and images for: Effect of chondroitin sulfate modified polyethyleneimine on mediating oligodeoxynucleotide YW002 in the treatment of periodontitis
Source: RSC Adv. 2024 Jun 25;14(28):20328–38. doi: 10.1039/d4ra00884g (PMC11197841; doi:10.1039/d4ra00884g)

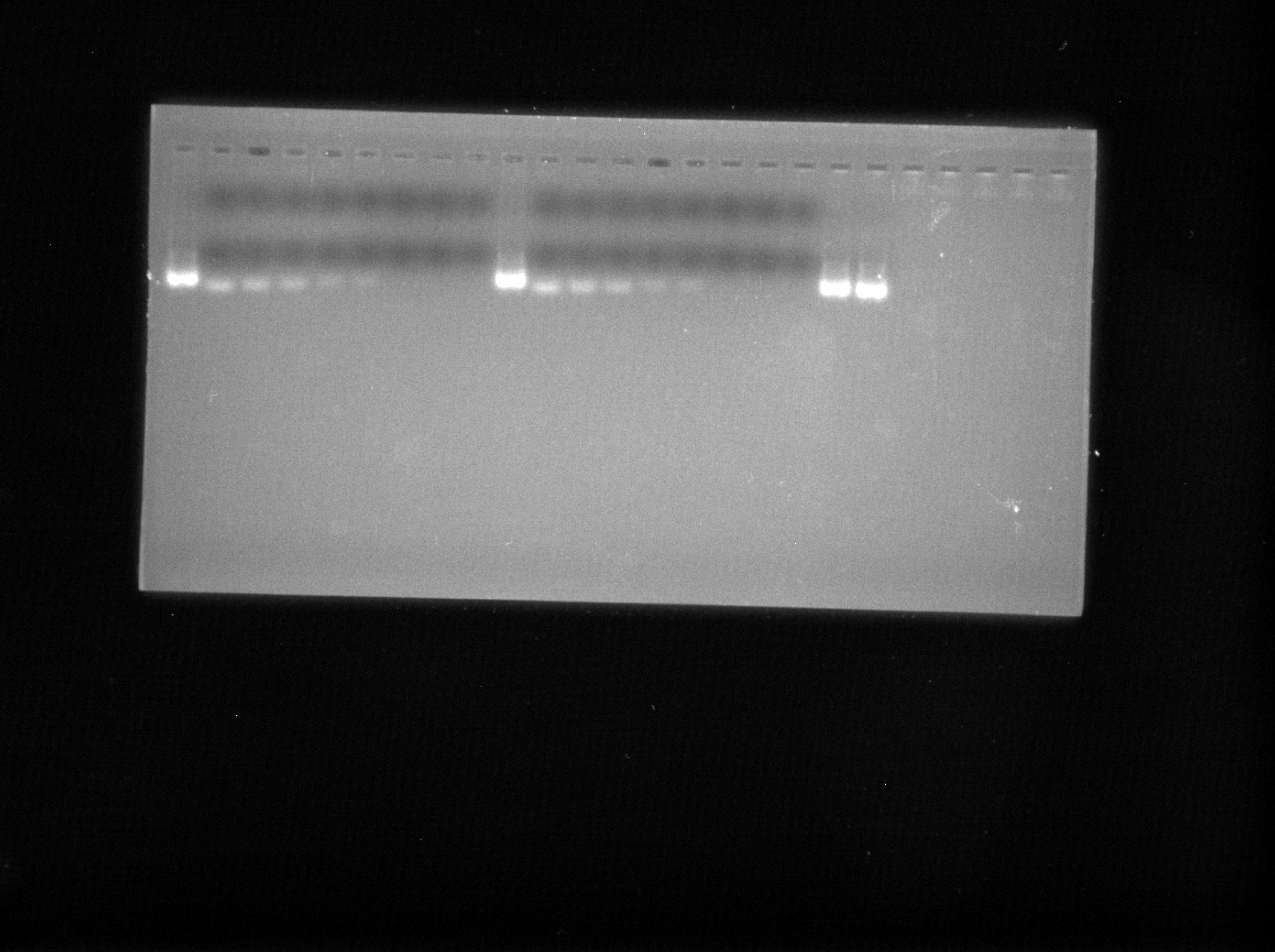

Supplement: RA-014-D4RA00884G-s002 [file RA-014-D4RA00884G-s002.zip › Original Images for Blots and Gels Requirements/Fig 2c Agarosegel retardation assays of PEI-CS YW002 nanocomplexes at different ww ratios.tif]

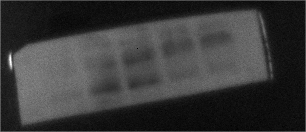

Supplement: RA-014-D4RA00884G-s002 [file RA-014-D4RA00884G-s002.zip › Original Images for Blots and Gels Requirements/Fig 4b IL-1a┬ 1.tif]

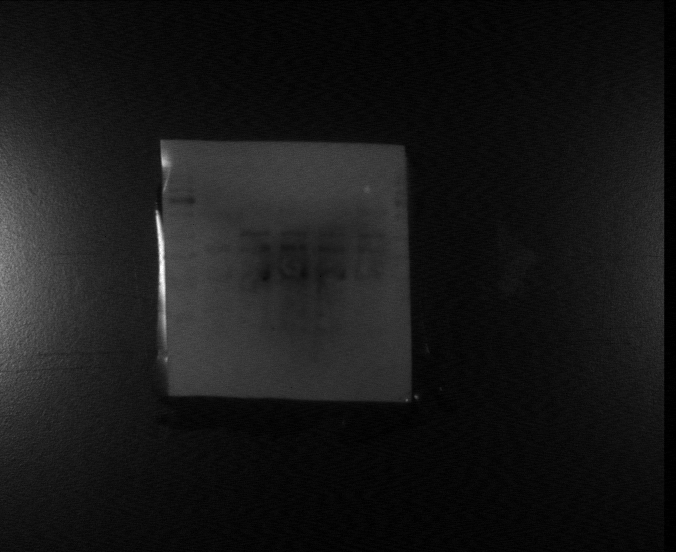

Supplement: RA-014-D4RA00884G-s002 [file RA-014-D4RA00884G-s002.zip › Original Images for Blots and Gels Requirements/Fig 4b IL-6.tif]

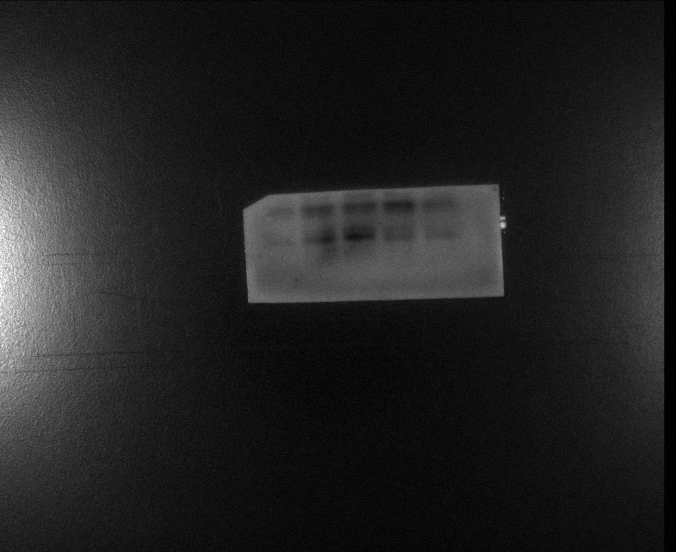

Supplement: RA-014-D4RA00884G-s002 [file RA-014-D4RA00884G-s002.zip › Original Images for Blots and Gels Requirements/Fig 4b TNF-a┴.tif]

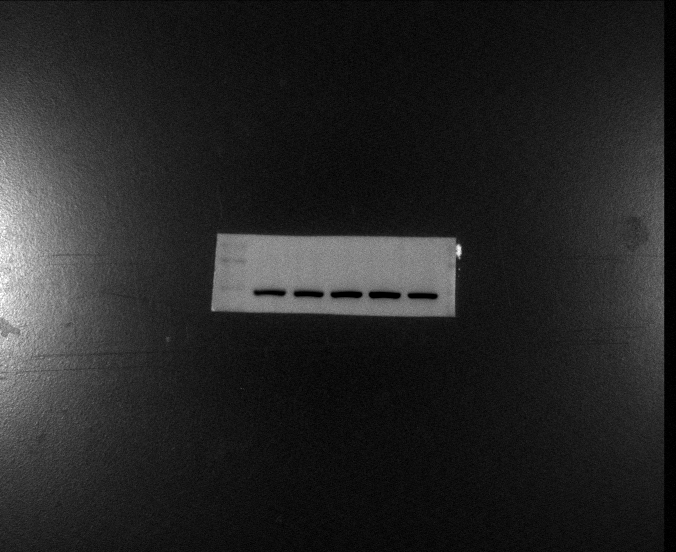

Supplement: RA-014-D4RA00884G-s002 [file RA-014-D4RA00884G-s002.zip › Original Images for Blots and Gels Requirements/Fig 4b a┬-actin.tif]

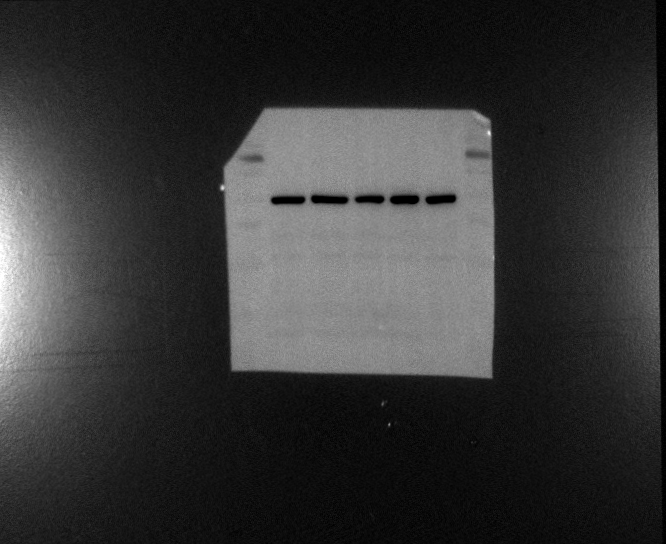

Supplement: RA-014-D4RA00884G-s002 [file RA-014-D4RA00884G-s002.zip › Original Images for Blots and Gels Requirements/IL-1a┬ 2 a┬-actin.tif]

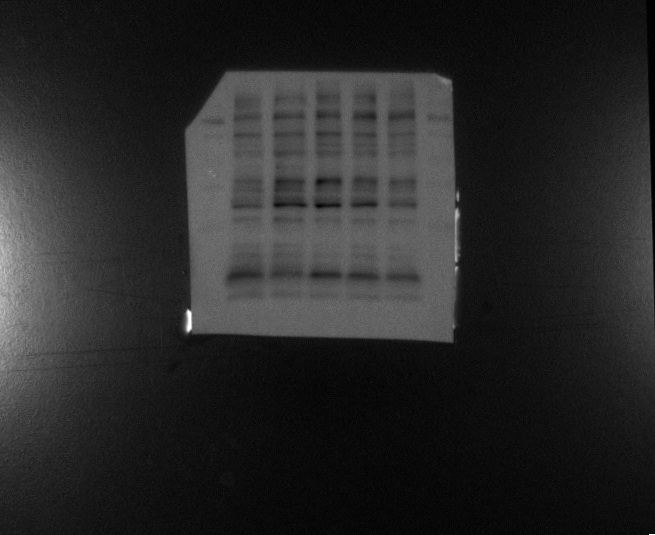

Supplement: RA-014-D4RA00884G-s002 [file RA-014-D4RA00884G-s002.zip › Original Images for Blots and Gels Requirements/IL-1a┬ 2.tif]

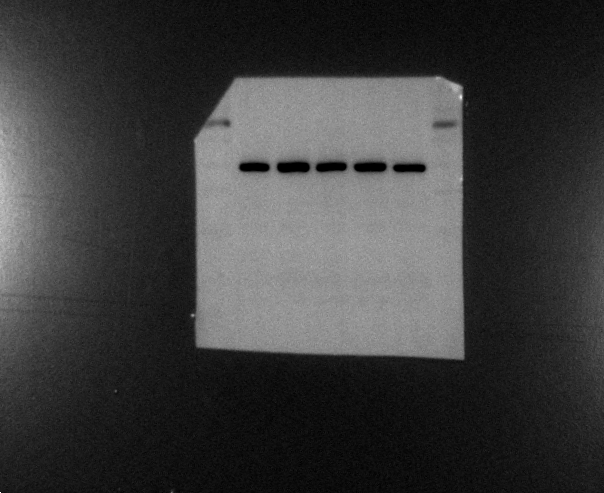

Supplement: RA-014-D4RA00884G-s002 [file RA-014-D4RA00884G-s002.zip › Original Images for Blots and Gels Requirements/IL-1a┬ 3 a┬-actin.tif]

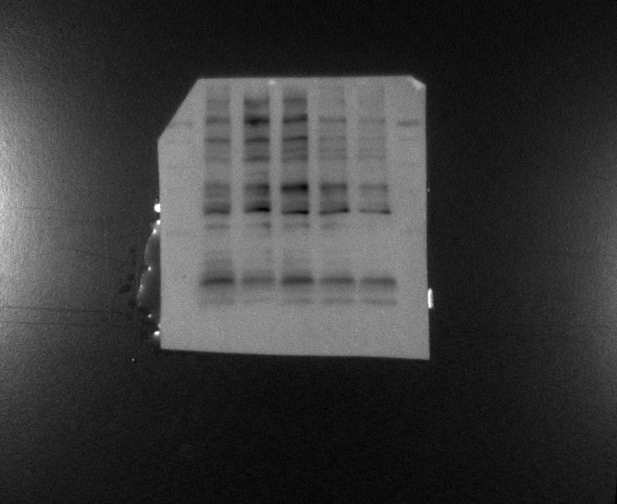

Supplement: RA-014-D4RA00884G-s002 [file RA-014-D4RA00884G-s002.zip › Original Images for Blots and Gels Requirements/IL-1a┬ 3.tif]

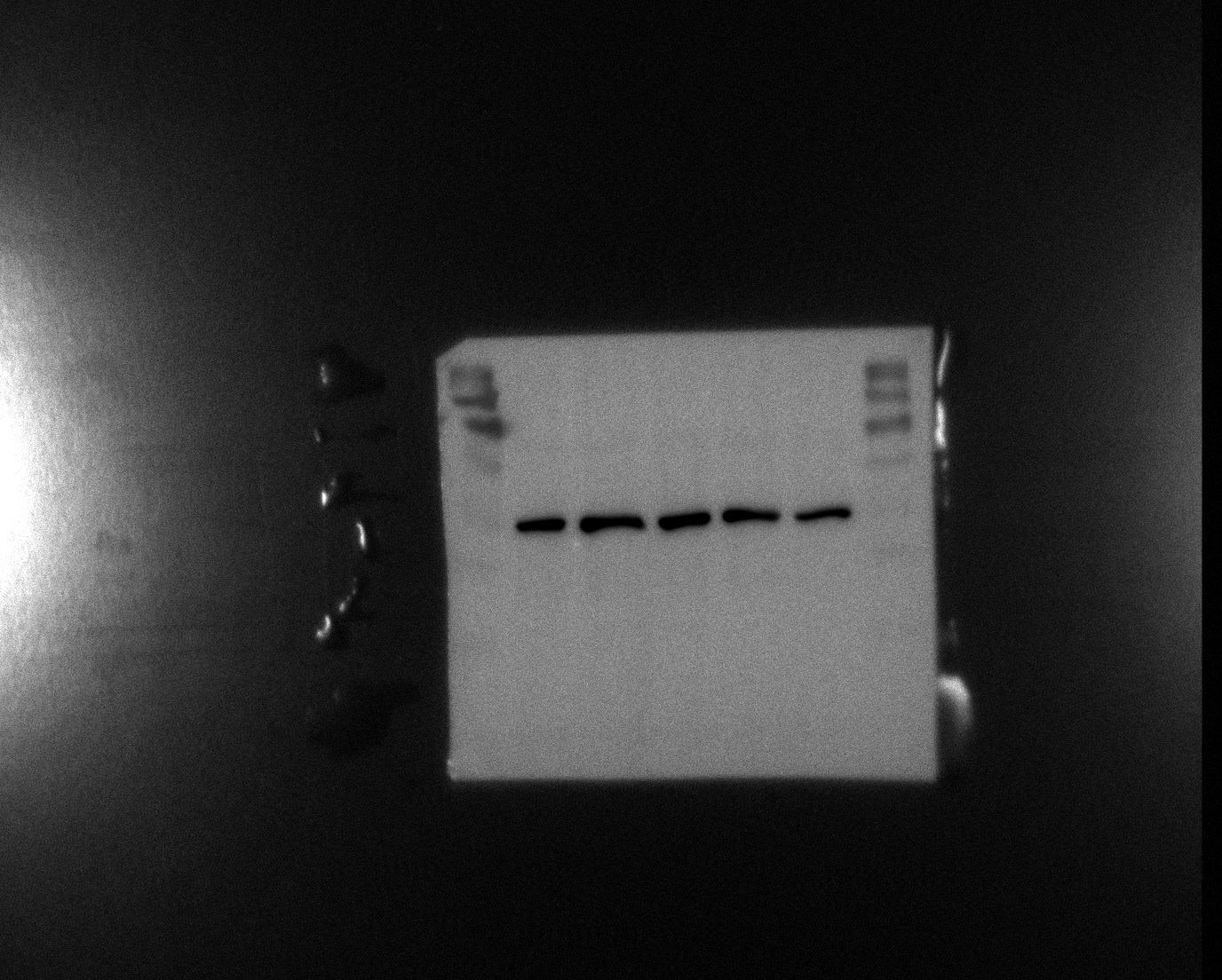

Supplement: RA-014-D4RA00884G-s002 [file RA-014-D4RA00884G-s002.zip › Original Images for Blots and Gels Requirements/IL-6 2 a┬-actin.tif]

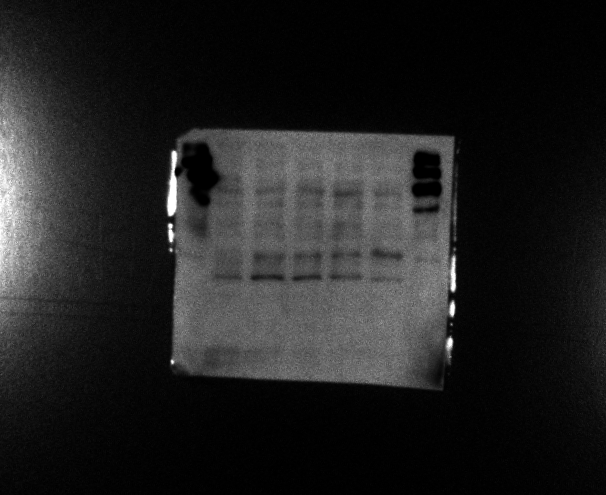

Supplement: RA-014-D4RA00884G-s002 [file RA-014-D4RA00884G-s002.zip › Original Images for Blots and Gels Requirements/IL-6 2.tif]

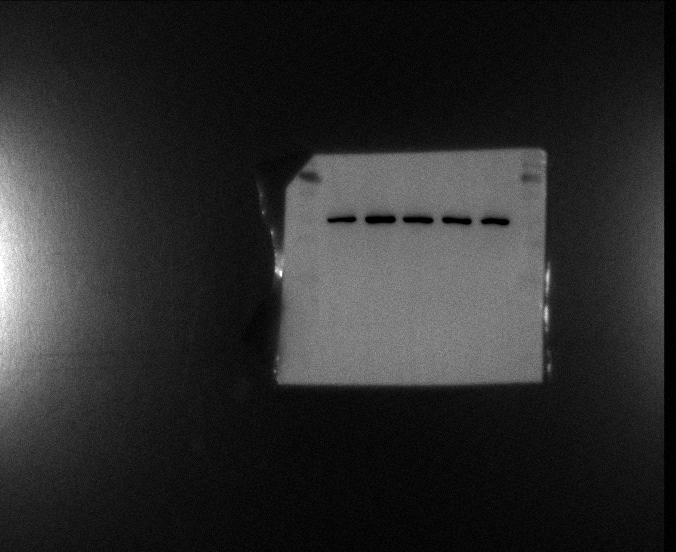

Supplement: RA-014-D4RA00884G-s002 [file RA-014-D4RA00884G-s002.zip › Original Images for Blots and Gels Requirements/IL-6 3 a┬-actin.tif]

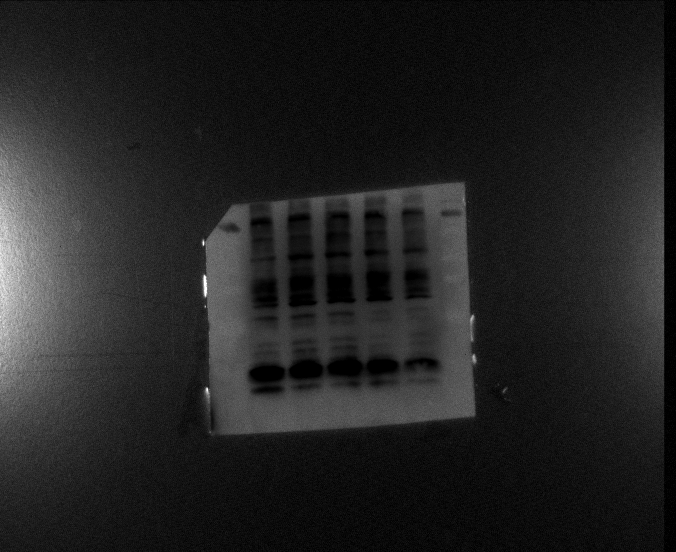

Supplement: RA-014-D4RA00884G-s002 [file RA-014-D4RA00884G-s002.zip › Original Images for Blots and Gels Requirements/IL-6 3.tif]

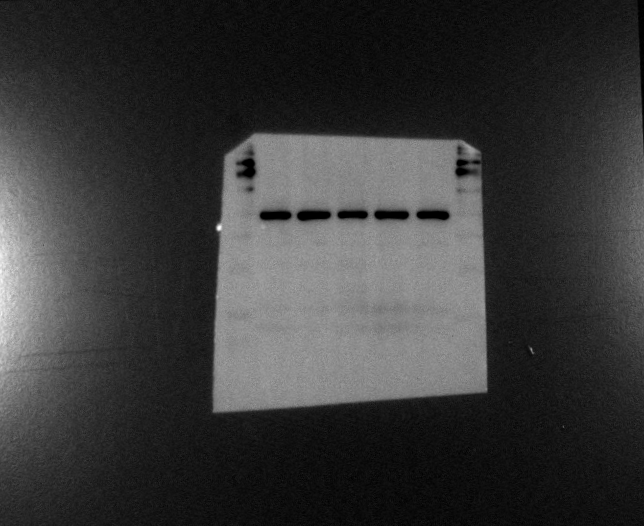

Supplement: RA-014-D4RA00884G-s002 [file RA-014-D4RA00884G-s002.zip › Original Images for Blots and Gels Requirements/TNF-a┴ 2 a┬-actin.tif]

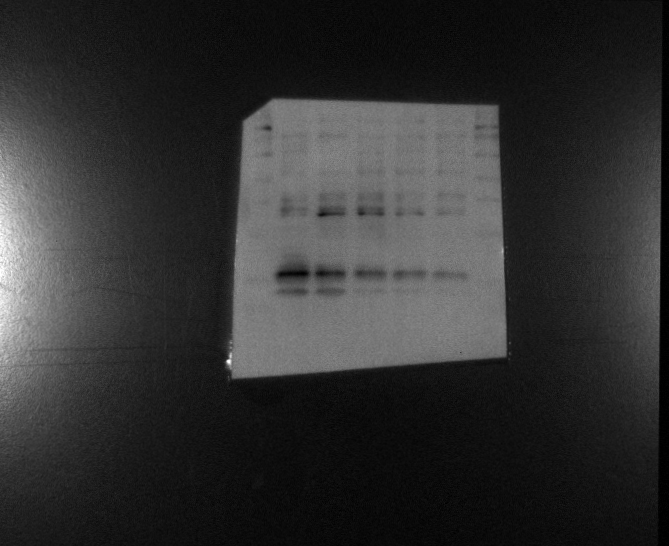

Supplement: RA-014-D4RA00884G-s002 [file RA-014-D4RA00884G-s002.zip › Original Images for Blots and Gels Requirements/TNF-a┴ 2.tif]

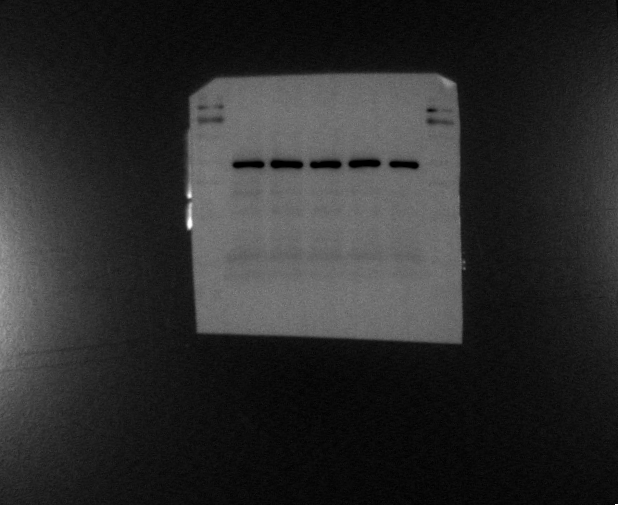

Supplement: RA-014-D4RA00884G-s002 [file RA-014-D4RA00884G-s002.zip › Original Images for Blots and Gels Requirements/TNF-a┴ 3 a┬-actin.tif]

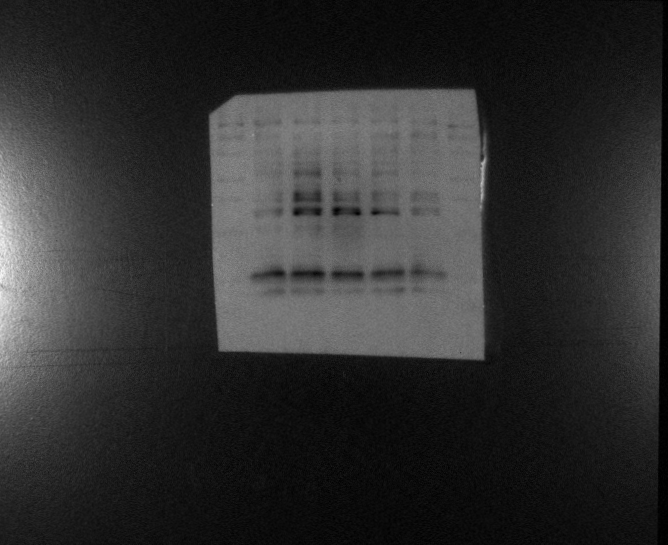

Supplement: RA-014-D4RA00884G-s002 [file RA-014-D4RA00884G-s002.zip › Original Images for Blots and Gels Requirements/TNF-a┴ 3.tif]
