# Supplementary material for: Effect of chondroitin sulfate modified polyethyleneimine on mediating oligodeoxynucleotide YW002 in the treatment of periodontitis
Source: RSC Adv. 2024 Jun 25;14(28):20328–38. doi: 10.1039/d4ra00884g (PMC11197841; doi:10.1039/d4ra00884g)
Supplement: RA-014-D4RA00884G-s003 [file RA-014-D4RA00884G-s003.pdf]

Electronic Supplementary Material (ESI) for RSC Advances.
This journal is © The Royal Society of Chemistry 2024

Materials and methods

Materials

YW002 (the sequence is: TCGCGAACGTTCGCCGCGTTCGAACGCGG) and YW002-s (YW002 modified by sulfur)、Cy5-labeled YW002 and YW002-s (TAKARA, China); Branched PEI (25 kDa) and CS (20 000 kDa) (Sigma-Aldrich, Germany); High glucose DMEM medium, Phosphate buffer solution (PBS) (Hyclone, USA), Fetal bovine serum (FBS) (Biological Industries, Israel), DAPI, BCA protein concentration determination kit (Beyotime, China), CCK-8 kit (Invigentech, USA), TRIeasy™ Total RNA Extraction Reagent, Hifair ® II 1st Strand cDNA Synthesis Super Mix for qPCR (gDNA digester plus)、Hieff ® qPCR SYBR Green Master Mix, Reverse Transcription Kit (Yeasen, China), Primer (Sangon, China), Polyvinylidene fluoride (PVDF) membrane (Millipore, USA),Blot loading buffer,ECL Chemiluminescence detection kit (NCM Biotech, China); antibodies against β-actin, IL-1β, IL-6 and TNF-α, horseradish peroxidase (HRP)-labeled goat anti-rabbit IgG and HRP-labeled goat anti-mouse IgG (Abcam, USA), P.g.-LPS (Solarbio, China).

Construction and characterization of PEI-CS

The CS solution was prepared by dissolving 1 g of CS in 50 mL of distilled water, followed by dropwise addition of varying amounts of methacrylic anhydride (MA). Subsequently, the pH of the reaction system was maintained at 8.0 by adding a 5 M NaOH solution. The reaction solution was stirred at room temperature for 2 h, followed by stirring at 8 ℃ for an additional 24 h. Subsequently, the reaction mixture was precipitated in ethanol and the resulting precipitate was collected through filtration to obtain the CSMA product. The reaction solution was stirred at room temperature for 2 hours, followed by stirring at 8 ℃ for an additional 24 h. Subsequently, the reaction mixture was precipitated in ethanol and the resulting precipitate was collected through filtration to obtain the CSMA product. The aqueous solution of CSMA (1 mg/mL) was added dropwise to PEI solution (1 mg/mL), and stirred at room temperature for 48 h.Afterwards,the product PEI-CS was purified by dialysis against distilled water for 48 h (molecular weight cut-off: 50,000 Da), and obtained by lyophilization. The PEI solution (1 mg/mL) was subjected to dropwise addition of an aqueous CSMA solution (1 mg/mL), followed by stirring at room temperature for 48 h. Subsequently, the resulting product, PEI-CS, underwent purification through dialysis against distilled water for 48 h using a molecular weight cut-off of 50,000 Da. Finally, the purified product was obtained via lyophilization. The structure of PEI-CS was characterized by 1H-NMR on an AVANCE DMX 500 spectrometer (Bruker,Germany) at 500 MHz in D2O at 25 ℃. The mean particle size and zeta potential of PEI-CS/YW002 nanocomplexes were determined by Malvern Nano ZS90 Zetasizer (Malvern,UK). The

morphologies of PEI-CS/YW002 nanocomplexes were examined using transmission electron microscopy (TEM) with a 200 KeV Talos F200s microscope (Thermo, USA).

Synthesize of YW002/PEI-CS nanocomplexes

Dissolve YW002 and PEI-CS with sterile purified water (pH 7.4) and dilute them to 10 μg/mL, mix evenly according to the mass ratio (0.25:1, 0.5:1, 1:1, 2:1, 4:1, 6:1, 8:1, w/w) of different solutes, and assemble at room temperature for 30 min, ready for use. The working concentrations of YW002 and YW002-s were 1 μg/mL.

Gel retardation assay

The PEI-CS/YW002 nanocomplexes were prepared by gently combining the carrier PEN and MT01 at various mass ratios (0, 1:1, 2:1, 4:1, 6:1, w/w) and allowed to incubate at room temperature for a duration of 30 minutes prior to utilization. The binding ability of PEI-CS/YW002 was evaluated through 1.5% agarose gel electrophoresis in a tris-acetate-EDTA (TAE) buffer solution (120 V, 20 min), and the results were documented using a gel imager (Bio-Rad, USA). To evaluate the protective effect of PEI-CS on YW002, the PEI-CS/YW002 nanocomplexes were incubated with 0.05 mg/mL RNase A and 50% FBS, respectively, and then the nanocomplexes were treated with heparin at a concentration of 10 mg/mL, and then detected by 1% agarose gel electrophoresis,

Cell culture and transfection

RAW 264.7 cells were grown to confluency in DMEM supplemented with 10% FBS, and were maintained at 37°C in a humidified atmosphere containing 5% CO2. Take RAW 264.7 cells incubated for 24 h, discard the solution, wash with PBS twice, and take YW002 (10 μg/mL)、YW002-s (10 μg/mL), PEI-CS/YW002 nanocomplexes with different solute mass ratios and PBS (blank control) were added into 6-well plates respectively, and high glucose DMEM medium (excluding FBS) was added to 2 mL (the final mass concentration of YW002/YW002-s in each well was 1 μg/mL), incubate with RAW 264.7 cells for 4 h, discard the solution, add high glucose DMEM medium (containing 10% FBS), and continue to incubate.

Cell viability assay

RAW 264.7 cells (5×10^3) were cultured in 96-well plates. PEI and PEI-CS/YW002 nanocomplexes solutions with different mass ratios incubate together at 37 °C for 4 h in high glucose DMEM medium (excluding FBS). Then discard the solution, add high glucose DMEM medium (containing 10% FBS), and continue to incubate for 24 h and 48 h according to the above-mentioned cell culture method. According to the manufacturer's instructions, the cytotoxicity of PEI-CS/YW002 nanocomplexes was analyzed by a CCK-8 kit and the absorbance was recorded at 450 nm by a microplate

reader (BioTek, USA). The cells were seeded in 6-well plates (2 × 10^5 cells/well) and incubated for 24 and 48 hours to facilitate live/dead cell staining. The fluid changes and incubation time followed the same protocol as the CCK-8 method. The live/dead cell dye was used for staining. Live cells were labeled green with calcein AM under excitation, while dead cells were stained red with propidium iodide (PI). The toxicity of PEI-CS/YW002 was observed and assessed using fluorescence microscopy.

CLSM

RAW 264.7 cells were seeded into confocal dishes and cultured according to the cell transfection method. The experiments were grouped as follows: control group, YW002 group, YW002-s group, and PEI-CS / YW002 group (4:1,w/w), YW002 and YW002-s were fluorescently labeled with Cy-5, YW002 and YW002-s working concentration were 1 μg/mL and incubated for 4 h. After that, samples were fixed with 1 mL of 4% paraformaldehyde for 15 min and stained with DAPI solution (10 μg/mL) for 5 min in the dark. CLSM used nuclear morphology of cells to observe endocytosis and uptake efficiency. Typically, nuclei are blue while YW002 and YW002-s are red. The quantification of fluorescence intensity was performed utilizing the Image J software (Media Cybernetics, USA).

RT-PCR

A total (2×10^5) RAW 264.7 cells were seeded in 6-well plates. Then cells were transfected according to the above-mentioned transfection method and stimulated with P.g.-LPS (5 μg/mL) for 24 h to establish an in vitro inflammation model. The experimental groups as follows: blank control group, LPS stimulation (inflammation) group, LPS + free YW002 group, YW002-s group, LPS + PEI-CS/YW002 group(4:1, w/w). The supernatant from the 6-well plates was aspirated and washed with PBS. Subsequently, 1 mL of TRIeasy Total RNA Extraction reagent was added to each well for the extraction of total RNA from cells in each experimental group. The purity and concentration of RNA were measured by a NanoDrop2000 spectrophotometer (Thermo,USA). The absorbance ratios at wavelengths of 260 and 280 nm ranged from 1.8 to 2.0. Reverse transcription was performed using the Hifair® II 1st Strand cDNA Synthesis Super Mix for qPCR reagent. An RT-PCR assay was performed using Hieff ® qPCR SYBR Green Master Mix on a Roche LightCycler 480 RT PCR Detection System (Roche, Switzerland) according to the manufacturer's protocol. All primers were designed and synthesized by China National Bioengineering Corporation (Shanghai, China). Gene expression levels were calculated using the expression $2^{-\Delta\Delta ct}$; The genes and primers are listed in **Table 1**.

Table 1. Primer sequence for qRT PCR.

Gene	Sequence of Primers (5′→3′)

β-actin	F:GGAGATTACTGCCCTGGCTCCTA<br>R:GACTCATCGTACTCCTGCTTGCTG
IL-1β	F:CCCTGAACTCAACTGTGAAATAGCA<br>R:CCCAAGTCAAGGGCTTGGAA
IL-6	F:AAGCCAGAGCTGCAGGATGAGTA<br>R:TGTCCTGCAGCCACTGGTTC
TNF-α	F:TTCCAATGGGCTTTCGGAAC<br>R:AGACATCTTCAGCAGCCTTGTGAG

Western blot analysis

The RAW 264.7 cells in five groups were cultured for 24 hours and subsequently lysed using RIPA buffer supplemented with 1% protease inhibitors. The protein concentration was determined utilizing the BCA protein assay kit. The protein samples were subjected to SDS-polyacrylamide gel electrophoresis, resulting in their separation, followed by subsequent transfer onto PVDF membranes. The membranes were blocked with blot blocking buffer and incubated overnight with primary antibodies: anti-IL-1β (1:500 dilution), anti-IL-6 (1:500 dilution), anti-TNF-α (1:500 dilution), anti-β-actin (1:2,000 dilution). The membrane was washed three times and then incubated with a secondary antibody (diluted 1:5,000) for an hour. The immunoreaction signals were detected using an ECL chemiluminescence detection kit and shown on a gel imaging system (Junyi, China).

Evaluation of periodontal inflammation reduction by PEI-CS/YW002 in vivo

Animal experiments were approved by the Animal Experimental Committee of College of Basic Medical Sciences, Jilin University, China (Ethical approval number: SYXK2023-0010). We used male C57BL/6 mice, 8 weeks old, average weight 18-22 g. Twenty-four male C57BL/6 mice were divided into four groups in a random manner: blank group (group I), periodontitis group (group II), periodontitis + YW002 treatment group (group III), and periodontitis + PEI-CS/YW002 treatment group (group IV). To establish a periodontitis model, the mice were anesthetized by inhalation of 1-1.5% isoflurane, and a nonabsorbable silk suture 4.0 was placed around the second molar of the maxilla of the mice for two weeks. On the day of establishing the periodontitis model, a volume of 20 μL YW002 or PEI-CS/YW002 was injected into the buccal-palatal gingival mucosa of the maxillary second molar on the experimental side in mice. Inject once every three days until the experiment ends, totalling five injections. The mice were anesthetized by intraperitoneal injection of 1% pentobarbital sodium after a period of 15 days, followed by euthanasia. The bilateral maxilla, and periodontal tissues of mice were taken for subsequent experiments.

Micro-CT

The mouse alveolar bone was fixed in 4% paraformaldehyde for 48 hours and fixed in a scanning container for CT scanning (SkyScan 1172; Bruker, Germany, 24 kV, 2 mA, 90 s). The alveolar bone level between the first and second molars was assessed in the sagittal plane. The three-dimensional images were meticulously reconstructed using the Materialise Mimics 21.0. The measurement of the distance between the cementoenamel junction (CEJ) and the alveolar bone crest (ABC) in each sample served as a standardized method for assessing alveolar bone resorption. The alveolar bone between the first and second molars was analyzed using bone parameter analysis software, encompassing measurements of bone volume fraction (Bv/Tv) and trabecular thickness (Tb.Th).

Histological Evaluation of Periodontitis in Mice with PEI-CS/YW002

The maxillae and vital organs collected from experimental mice were fixed in 4% PFA and decalcified in 0.5 M EDTA for a duration of 4 weeks for histological analyses. After decalcification, the tissues underwent dehydration, embedding, and sectioning. Sections were cut along a long axis of first premolar teeth, with a thickness of approximately 3 μm. The periodontal tissue of mice was assessed for inflammation through H&E staining. The CEJ-ABC distance was measured as an indicator of alveolar bone resorption.

Anti-Inflammatory Properties of PEI-CS/YW002 nanocomplexes In Vivo

Total RNA from mouse maxillary alveolar bone preserved in liquid nitrogen was extracted with TRIzol and amplified by reverse transcription-polymerase chain reaction (qRT-PCR).

Statistical analysis

SPSS v23.0 software (IBM, Armonk, NY, USA) and Prism GraphPad v9.0 software (La Jolla, CA, USA) were used for data analysis. All data were normality checked and expressed as mean ± standard deviation (SD). The multi-sample measurement data were compared using the Single Factor ANOVA Tukey's method. $p<0.05$ was considered as significant difference.

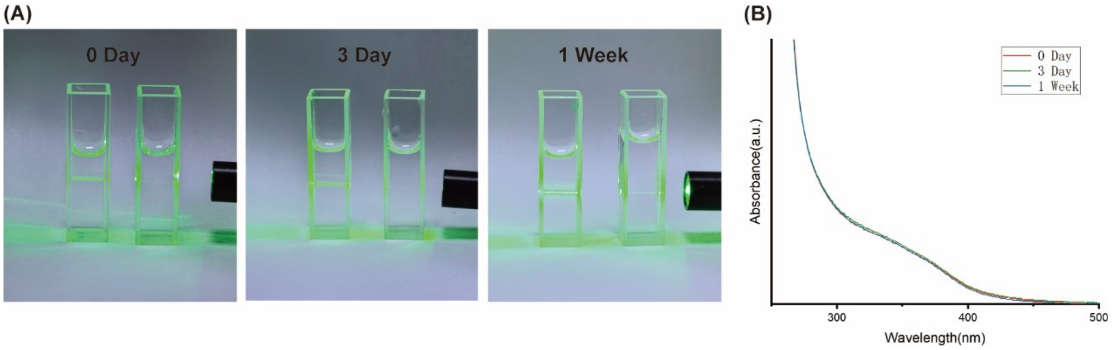

Supplementary Figures

Figure S1. (A) Tyndall effect of PEI-CS/YW002 nanocomplexes at different time points (The right cuvette contained deionized water and the left cuvette contained PEI-CS/YW002 nanocomplexes. (B) UV-vis spectra of PEI-CS/YW002 nanocomplexes at different time points.

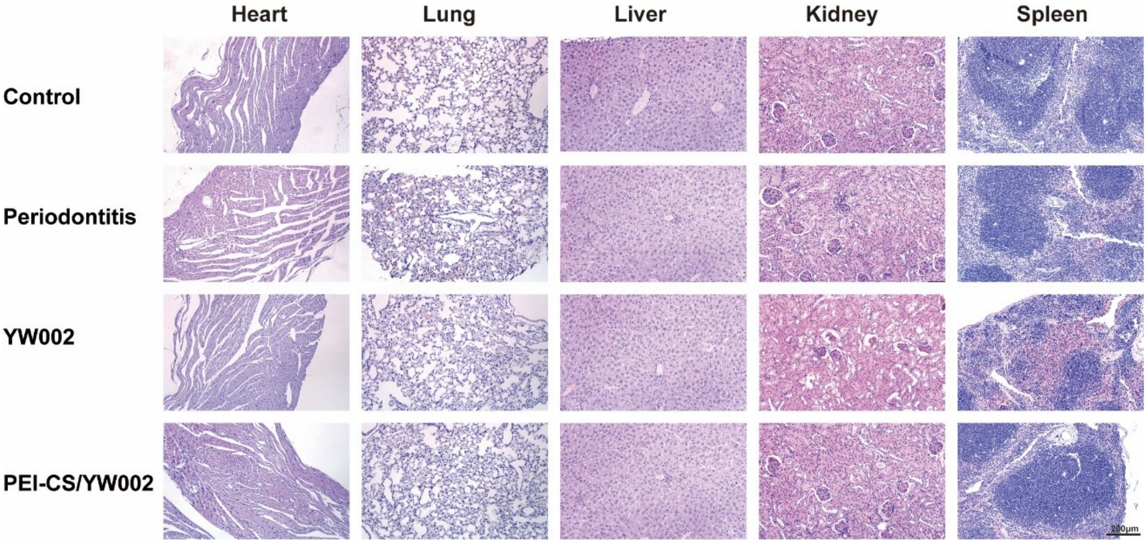

Figure S2. Biocompatibility of PEI-CS/YW002 nanocomplexes in female mice in vivo.
